# Supine positioning after vitrectomy for rhegmatogenous retinal detachments with inferior retinal breaks

**DOI:** 10.1186/s40942-020-00247-8

**Published:** 2020-09-14

**Authors:** Amr Mohammed Elsayed Abdelkader, Hossam Youssef Abouelkheir

**Affiliations:** 1 Department of ophthalmology, Lecturer of ophthalmology, Mansoura ophthalmic center, faculty of medicine, Mansoura university, Mansoura, Egypt; 2 Assistant professor of ophthalmology, Mansoura ophthalmic center, faculty of medicine, Mansoura university, Mansoura, Egypt

**Keywords:** Face up position, Pars plana vitrectomy, Retinal breaks, Rhegmatogenous retinal detachment, Silicone oil

## Abstract

**Background:**

To evaluate the effectiveness of face up position (FUP) following pars plana vitrectomy (PPV) and silicone oil injection in cases of rhegmatogenous retinal detachment (RRD) with multiple peripheral and inferior breaks.

**Method:**

Thirty-two eyes of 32 patients with RRD due to multiple peripheral breaks were managed with PPV and silicone oil as endotamponade. Postoperatively, all patients were instructed to assume face up (supine) position for at least 10 days. Silicone oil was removed 3 to 6 months postoperatively in eyes with attached retina and the patients were followed up for 6 months.

**Results:**

Thirty eyes (94%) got a successful attachment of the retina and remained attached after silicone oil removal. One case showed lower redetachment under silicone oil while the other case showed recurrent RRD after silicone oil removal.

**Conclusion:**

Although postoperative FUP is not a popular one, it is effective in the treatment of RRD with peripheral breaks whatever the number or the distribution of these breaks. This may in some way or another change the traditional trends of postoperative positioning after vitrectomy for RRD.

## Background

Silicone oil is a synthetic polymer that proved to be a valuable tool in the treatment of rhegmatogenous retinal detachment (RRD). Owing to its low specific gravity (0.97 g/ml), the fluid in the vitreous cavity goes down and the silicone bubble floats upwards [1]. Postoperatively the patient is positioned in a way that makes the surface of the bubble close the retinal breaks. Face down position (FDP) remains a common decubitus described to the patients after vitrectomy [2–4] Actually, this is of limited value as the main function of the intraocular tamponading agent is to occlude retinal breaks till firm adhesion is created by retionpexy and the remaining retina will flatten even if a residual subretinal fluid is present [5]. So, it is more important to tamponade or plug the breaks rather than to push the retina backwards towards the posterior pole of the eye. An ideal position to achieve this goal is to direct the patient to lay supine with his face up as long as there is no posterior breaks.

The aim of this work is to determine whether maintaining FUP after PPV and silicone injection for RRD with peripheral breaks would be beneficial or not.

## Patients and methods

This study was conducted in Mansoura university ophthalmic center from August 2016 to October 2019. Thirty-two eyes of 32 patients with RRD due to multiple peripheral breaks located in both temporal and nasal halves with at least 1 causative break located inferiorly between 4 and 8 o’clock were included in this study. Exclusion criteria were: RD with posterior retinal breaks, RD with proliferative vitreoretinopathy grade C or more, aphakia and eyes with anterior chamber intraocular lens (ACIOL).

All cases underwent 23 gauge pars plana vitrectomy (PPV) by one of the two surgeons (Hossam Abouelkheir and Amr M Abdelkader). After insertion of the 23 gauge cannulas, the vitrectomy cutter was used to remove the central vitreous and to induce detachment of the posterior hyaloid. Posterior hyaloid detachment was assured with the aid of triamcinolone acetonide and any residual layer of attached posterior cortical vitreous was removed. Perfluorocarbon (PFC) liquid was injected to flatten the posterior retina and direct the subretinal fluid anteriorly. Meticulous shaving of vitreous base was done with the aid of scleral indentation and the retinal breaks were identified and marked with endodiathermy. Fluid air exchange was then performed and the subretinal fluid was carefully drained through the accessible peripheral retinal breaks or induced peripheral retinotomy. After complete filling of the vitreous cavity with air, two rows or more of laser photocoagulation were applied around retinal breaks and extended to include 360^o^ of the retinal periphery. Before injection of silicone oil, any reaccumulating fluid over the posterior retina was removed to assure good filling of the vitreous cavity with silicone. In cases with lenticular opacity that prevented adequate visualization of the posterior segment, phacoemulsification with foldable PCIOL implantation was performed combined with vitrectomy.

Postoperatively, all cases were instructed to maintain FUP (supine) for 10 to 14 days. The patients were directed to strictly maintain the position during the first 6 to 8 h after surgery and then as long as possible throughout the following days. The patients were examined during the first postoperative day, weekly for one month and monthly until the time of silicone oil removal (3–6 months). After silicone oil removal the patients were followed up for another 6 months.

## Results

The age of patients ranged from 21 to 63 years old (mean 38.72). Twenty-two eyes were phakic and 10 eyes were pseudophakic with PCIOL. The crystalline lens was clear in 14 eyes, while 8 eyes showed variable degrees of cataract. Combined PPV and phacoemulsification with PCIOL implantation were performed in 4 cases.

The macula was flat in two cases. During surgery, peripheral iatrogenic retinal holes occurred in 4 cases and peripheral retinotomy were done in 3 cases. The final number of retinal breaks (including iatrogenic holes and retinotomies) was 3 in 14 eyes, 4 in 12 eyes, 5 in 4 eyes and 6 in 2 eyes as shown in Table 1. No direct lens injury or other significant complications were encountered during surgery.

**Table 1:** distribution of the Preoperative BCVA, Post operative BCVA after 3 months, No of retinal breaks, No and distribution of affected quadrants

Case no.	Preoperative VA (Average) 1.65 Log MAR units	Postoperative VA (Average) 0.85 log Mar units	No of retinal breaks	NO quadrants affected
1	2.3	1.3	6	4
2	1.3	0.5	4	4
3	2.3	1.3	3	3 (Inferior, Superior and nasal)
4	1.8	0.4	3	4
5	0.9	0.5	3	2 (Inferior and superior)
6	2.3	1.3	4	2 (Inferior and nasal)
7	1.3	0.6	3	2 (Inferior and nasal)
8	1.3	0.6	3	4
9	2.3	1.3	3	2 (Inferior and temporal)
10	1.8	1.3	4	4
11	1.8	0.5	5	3 (Inferior, Superior and nasal)
12	2.3	1.3	3	3 (Inferior, Superior and temporal)
13	2.3	1.3	3	3 (Inferior, Superior and temporal)
14	1.3	0.5	3	2 (Inferior and temporal)
15	1.3	0.6	3	4
16	1.3	0.8	4	2 (Inferior and temporal)
17	1.8	1	4	3 (Inferior, Superior and temporal)
18	2.3	1.3	6	3 (Inferior, Superior and nasal)
19	1.6	0.8	5	4
20	1.3	0.5	4	3 (Inferior, Superior and temporal)
21	1.8	1	4	3 (Inferior, Superior and nasal)
22	1.3	0.6	5	3 (Inferior, Superior and temporal)
23	1.8	1	4	4
24	1	0.5	3	2 (Inferior and nasal)
25	1.8	1.3	4	3 (Inferior, Superior and temporal)
26	1.3	0.6	3	2 (Inferior and temporal)
27	1.6	1	5	3 (Inferior, Superior and temporal)
28	1.8	1	4	4
29	1.3	0.5	3	2 (Inferior and temporal)
30	1.3	0.6	4	2 (Inferior and nasal)
31	1.3	0.4	4	3 (Inferior, Superior and Nasal)
32	1.8	1.3	3	4

Primary retinal reattachment occurred in 30 cases (94%) while redetachment occurred in 2 cases. One case showed recurrent inferior retinal detachment while silicone oil was inside the eye. The recurrence was noted 4 weeks after surgery. The patient relatives recorded reluctance of their patient to maintain the postoperative positioning. After discussion with the patient, re-operation was done and retinectomy to the inferior peripheral retina was performed, followed by new laser application and heavy silicone oil (Densiron ^68 R^, Dorc) injection. Densiron was removed after approximately 2 months and the retina remained attached. The other case showed recurrent retinal detachment after silicone oil removal (6 months after primary surgery). Recurrence occurred due to a missed hole which was observed during re-operation just posterior to the previously applied laser nearly at 12 O’clock. During re-operation the eye was left filled with air which was enough to achieve successful re-flattening of the retina.

The mean pre-operative log MAR best corrected visual acuity was 1.65 (SD ± 0.71), while the post-operative one was 0.85 (SD ± 0.39) as shown in Table 1. This improvement was statistically significant (p < 0.0001). The range of visual acuity preoperatively was: hand movement to 6/9 and postoperatively it was: 1/60 to 6/9. Before surgery the mean intraocular pressure (IOP) was 10.38 (SD ± 1.8 mmHg) and raised to 15.41 (SD ± 3.23 mmHg) 1 month after vitrectomy. Three cases showed elevation of the IOP during the first week after surgery which necessitated the use of antiglaucoma medications. All these 3 cases had undergone combined phacoemulsification and PPV. The elevated IOP was supposed to be due to either residual viscoelastic materials or inflammatory reaction. The IOP gradually decreased in 2 cases and the anti-glaucoma medications were discontinued. The third case needed continuous use of topical anti-glaucoma (Timolol and Drozolamide in a combined form) even after removal of silicone oil.

Silicone oil escape under the conjunctiva was noticed in one case and its migration into the anterior chamber (AC) occurred in another case. The silicone bubble in the AC was discovered in the first postoperative day in a previously pseudophakic patient with broken posterior capsule and slightly laterally displaced PCIOL. The bubble in AC was selectively removed 3 weeks later. The retinal condition remained stable in the previous 2 cases throughout the follow up period.

Epimacular membranes with macular puckering developed in 2 cases (6.2%) and where noted nearly two months after primary surgery. These 2 cases were directed for early silicone oil removal (3 months after vitrectomy). During silicone oil extraction these membranes were successfully removed together with internal limiting membrane peeling. Table 2 summarizes the postoperative complications.

**Table 2:** postoperative complications

Complications	No. of cases (%)
Elevated IOP	3 (9.4)
Cataract	6 (43)
Silicone escape under conjunctive	1 (3.1)
Silicone in A.C	1 (3.1)
Epimacular membrane	2 (6.2)
PVR	0
Recurrence under silicone	1 (3.1)
Recurrence after silicone removal	1 (3.1)

*IOP* Intraocular pressure, *AC* Anterior chamber, *PVR* Proliferative Vitreoretinopathy

## Discussion

The presence of multiple and inferior retinal breaks is a challenge for choosing the proper tamponade and the most effective postoperative positioning. Heavier than water tamponades and purified liquid perfluorocarbons have been used for better inferior retinal tamponading. However, they are not effective against upper retinal breaks, more expensive than standard silicone oil, carry more risk of intraocular inflammation and IOP rise and necessitates prompt removal and more strict follow up [6]. So, the preferable choice for many vitreoretinal surgeons in cases with RRD is Pars plana vitrectomy with intraocular injection of a standard tamponading agents either silicone oil or gas. Postoperatively, the patients were positioned in a way that makes the retinal breaks uppermost to provide the adequate effect of the floating tamponading material. Difficulties appear when multiple retinal breaks are present and located on opposite sides of the vertical meridian or when retinal breaks are located inferiorly. In these cases, face down (prone) position (FDP) was traditionally considered as an ideal solution [7, 8]. However, maintaining FDP for many days after surgery is very annoying for most patients, especially children, elderly, obese persons, cardiac patients and those with vertebral column troubles [9]. Also, the patient communication with his family and surrounding is difficult which add psychic stress. It will be interesting to provided the patient with an alternative postoperative position (FUP) that could provide the same target.

**Fig. 1:**
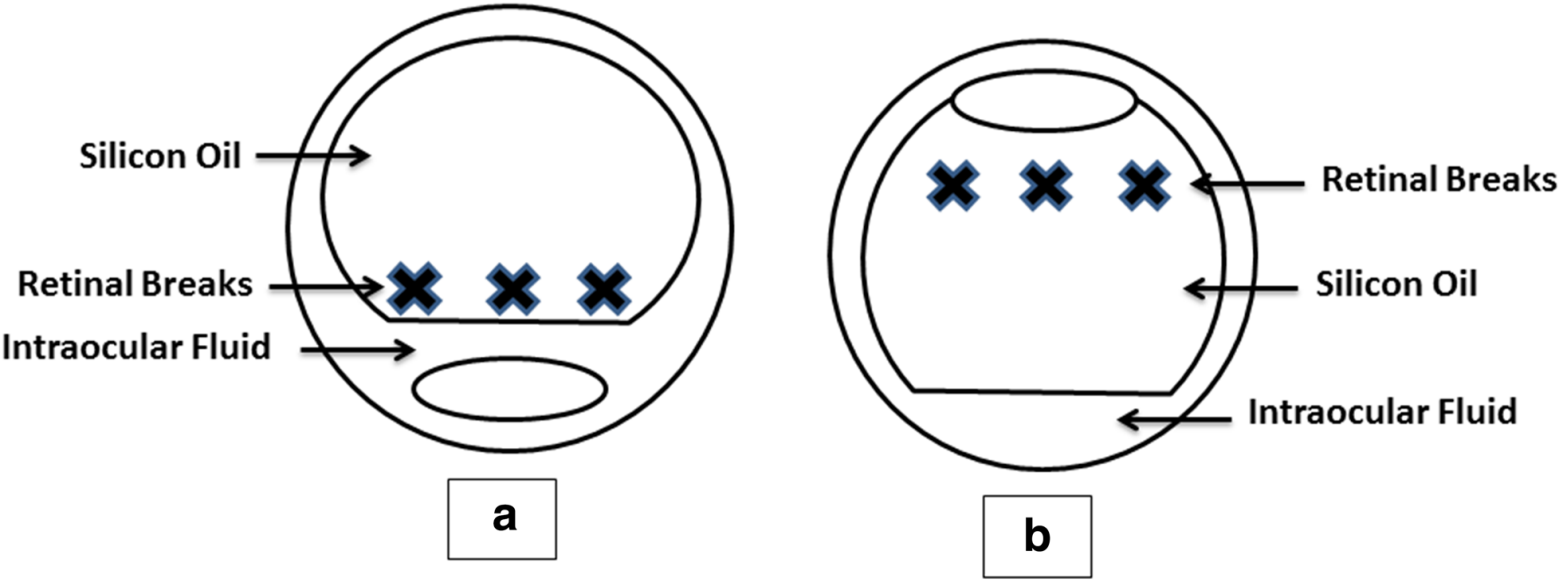
A diagrammatic representation of the silicon tamponade position in different head postures: **a** shows silicon filled eye during face down position and **b** shows silicon filled eye during face up position

On assuming FDP position, any aqueous fluid will be displaced to the retro-lental space away from retinal periphery [7]. While during FUP position, the aqueous fluid in the vitreous cavity will be displaced posteriorly to the premacular area allowing the tamponading agent to float anteriorly securing all the peripheral breaks adequately. In fact, during FUP the aqueous fluid is far away from the peripheral breaks if compared with FDP (Fig. 1).The previous relation can be watched out during the last steps of vitrectomy after fluid-air exchange. While the patient is laying supine in the operating room, the residual vitreal and sub retinal fluid, always goes posteriorly while air fills the remaining vitreous cavity maintaining the peripheral retina attached and the retina never redetach intra-operatively. This also remains the same when silicone oil is injected. Really, it looks strange to ask the patient to change this operative secure supine (FUP) to the uncomfortable prone (FDP) postoperatively.

More Over, The ability of the patient to comply with FDP varies greatly. Seno et al. [10] studied compliance with face down position during the first 3 days after vitrectomy. Only 30% of patients had perfect compliance. Patients who had been unable to comply were mostly males and this was obvious during deep sleep. Another study showed that the real time of face down position after surgery was around 5.5 h per day [11]. Feist et al. [12] reported that the non supine position is preferred by patients over FDP after macular hole surgery. 49% of the cases refused to repeat the experience of face down positioning if another surgery is mandatory. Many efforts have been made to decrease physical and psychological burdens during FDP such as; supporting devices [13–15], spectacle mounted prisms [16], and body message [17]. On the contrary, no special devices are needed during FUP and the position can be maintained easily during sleep. Also, the installation of topical drugs doesn’t interfere with FUP unlike FDP [18].

No previous study, to the best of our knowledge, was designed to evaluate the effectiveness of strict FUP in RRD cases with multiple quadrants and inferior retinal breaks. The current study was the first study designed to evaluate the effect of strict post operative supine position after vitrectomy for retinal detachment with multiple quadrants and inferior retinal breaks. Few studies have mentioned the use of FUP combined with other postures (non prone position) in the treatment of RRD. Those studies were based on the physical effect of intraocular tamponade, i.e., the surface tension not the buoyancy, which precludes the retinal breaks and prevent the intraocular fluid to escape into the subretinal space [19, 20]. Especially when coupled with creating adequate chorioretinal adhesion by complete drainage of subretinal fluid and sufficient photocoagulation [21]. Besides, on anatomical basis, the intraocular tamonade might not come into sufficient contact with inferior retinal breaks during prone positioning. Furthermore, Bell’s phenomenon in prone position moves the intraocular tamponade away from inferior retinal breaks. Consequently, our assumption that post operative face up is a novel approach for tamponading inferior retinal breaks. Sharma et al. [22] used either FUP or cheek down posture for one week after vitrectomy in cases with RRD due to inferior breaks and the success rate was 81.3%. In another study, Chen et al. [9] compared strict FDP for at least 7 days postoperatively with adjustable positioning. In the adjusted group, the postoperative positioning depended on the location of retinal tears. FUP was only described in few pseudophakic eyes. Retinal reattachment after primary surgery occurred in 89.7% of cases in the FDP group and 92.7% in the adjusted group. Similar study was done by Shiraki et al. [23] in 2018, that compared vitrectomy with and without prone positioning for rhegmatogenous retinal detachments in eyes with inferior retinal breaks. This study included six eyes in the prone position group while eighteen eyes were included in the group without prone positioning. The initial reattachment rate in eyes with inferior retinal breaks without postoperative prone positioning was 94.7%, which was significantly better than the just 60% in the group with postoperative prone positioning (p value: 0.036). The BCVAs were similar between the two groups after the 3-month postoperative. So Shiraki et al. [23] stress on the efficacy of not maintaining a prone position after vitrectomy to manage RDs with inferior breaks. However, The Shiraki et al. study had some limitations including small sample size and the areas of the retinal detachment were greater in the prone position group, which results in lower anatomical success rate. In our study primary retinal reattachment was achieved in 94% of case. The use of silicone oil (not gas) and maintaining the position for longer duration (10 days at least) may contribute to this high success rate.

In the current study, the mean pre-operative log MAR best corrected visual acuity was 1.65 (SD ± 0.71), while the post-operative one was 0.85 (SD ± 0.39). The mean post-operative improvement was statistically significant (p < 0.0001). Afrashi et al. [2] performed vitrectomy with silicone oil injection and postoperative prone position for 22 eyes with multiple breaks. The mean post-operative improvement in visual acuity was log MAR 0.7 ± 0.9 while in our study it was 0.8 ± 0.46. The relatively better value in our work may be attributed to the larger pre-operative number of cases with detached macula 30/32 (93%) compared to 14/22 (64%) in their study.

We didn’t have any case of pupillary block glaucoma (PBG) in our study. The zonules and the lens capsule act as a barrier preventing anterior migration of silicone oil in phakic and pseudophakic eyes. Jackson et al. [24] and Ragab et al. [25] stated that silicone bubble can migrate into the anterior chamber if there is a dehiscence in the zonules or a posterior capsular opening. Al Jazzaf el al [26] studied the incidence and features of glaucoma after vitrectomy and silicone oil injection in 450 eyes. They found that even when anterior migration of silicone oil occurs this doesn’t necessarily lead to the development of PBG. In our study, silicone bubble escaped into the anterior chamber in 1 case. The small size of the bubble and the routine post-operative instillation of cycloplegic drops may have helped to prevent PBG.

Some studies have reported a displacement of inflammatory and pigment epithelial cells to the inferior retina after vitrectomy because of the effect of gravity, which increase the incidence of development of lower proliferative membranes [27, 28]. These cells may be expected to gravitate on the macular area with maintaining face up for nearly 2 weeks. However, no increased incidence of epimacular membranes was noticed in this study. Epimacular membranes only developed in 2 cases out of 32 eyes (6.25%). The recorded incidence of macular puckering after PPV for repair of RRD varies from 3 to 12,8% [29, 30]. These two cases were easily treated with epimacular membranes removal and internal limiting membrane peeling during silicone oil removal.

Macular folds were not noticed in any case post-operatively. Meticulous drainage of subretinal fluid together with the use of PFC liquid allowed minimal amount of subretinal fluid to escape under the macula. This amount was absorbed by RPE with smooth reattachment of the macula. Heinmann and Bopps [31] stated that there is an argument regarding the best postoperative position to decrease the incidence of macular folds. The floatation force of the gas bubble during face down position may promote flattening of the macula with no folds. However, this force may form and fix a fold in the macular area. They mentioned the possible value of supine (FUP) position during the first hours after surgery in minimizing the risk of macular folds.

The ocular limitations for FUP include the presence of posterior breaks or retinotomies. Clinical practice reveals that posteriorly located breaks are rarely encountered in RRD, also the use of PFC liquids nullified the need of posterior retinotomy for drainage of subretinal fluid. Aphakic patients may be a relative contraindication for FUP as silicone oil may migrate into the anterior chamber. The general limitations include patients with cardiac troubles or respiratory diseases who can’t lay flat. Also, some obese patients may find difficulty on assuming this posture.

our results using silicone oil were comparable to the study conducted by Vicente Martinez-Castillo et al. They operated on 147 pseudophakic eyes with primary RRD with inferior retinal breaks with initial success in 139 cases (94.5%), however six cases (4.1%) re-detached because of incomplete retinal adhesion of the treated breaks, 61% of cases (91 cases) were presented with a single break only and the macula was de-attached in 80% of cases (118 cases) [32]. We suppose that gas can also tamponade the peripheral breaks in the FUP but two points should be taken into consideration. First, gases in non-expansible concentrations gradually decrease in size in the postoperative period, allowing aqueous to refill the vitreous cavity. This will necessitate more strict positioning and may carry the risk of redetachment. Second, the gas bubble in phakic eyes with its high floatation force excretes high pressure on the crystalline lens compared to silicone oil. The buoyancy of 1 ml of gas in aqueous equals 1 gm. With the same volume of silicone oil it markedly decreases to 0.03 gm [33]. Therefore, assuming FUP in gas filled eyes will lead to the formation of irreversible, progressive lens opacity contrary to the reversible feathery cataract noticed in gas filled eyes with post-operative FDP. Chen et al. [9] used long acting gas (C3F8, 14%) with FUP only in pseudophakic patients. So, FUP can be limited to pseudophakic eyes or accompanied with phacoemulsification and intraocular lens implantation when gas is planned to be used. However the exact efficacy and the risks of using gases with FUP have to be evaluated in another work as silicone oil was used for tamponading in all cases in this study.

The limitations of this study include lack of randomization with other conventional approaches, namely facedown positioning with gas or silicone oil tamponade with or without a scleral buckling in addition to the relatively limited sample size.

In summary, FUP could be a universal position for all peripheral breaks. In assuming this position, 360^°^ circumference of the retinal periphery is adequately supported at the same time. This will be ideal for cases with multiple, inferior and undetected breaks. Since both FUP and FDP are effective, we may give our patients the option to choose between the two positions giving a chance for better compliance. The possibility of using alternating FDP and FUP postoperatively may be another option but needs a separate evaluation in another study.
